# Unleashing the power of AI in predicting the technological and phenolic maturity of pomegranates cultivated in Lebanon

**DOI:** 10.1038/s41598-025-01936-w

**Published:** 2025-05-30

**Authors:** Rim Ghannoum, Nourhan Taha, David D. Gaviria, Hiba N. Rajha, Nada El Darra, Shadi Albarqouni

**Affiliations:** 1 Department of Nutrition and Dietetics, Faculty of Health Sciences, Beirut Arab University, Tarik El Jedidah, Riad El Solh, P.O. Box 115020, Beirut, 1107 2809 Lebanon; 2 Clinic for Diagnostic and Interventional Radiology, University Hospital Bonn, Venusberg-Campus 1, 53127 Bonn, Germany; 3 Département de Génie Chimique et Pétrochimique, Faculté d’Ingénierie, Ecole Supérieure d’Ingénieurs de Beyrouth (ESIB), Université Saint-Joseph de Beyrouth, CST Mkalles Mar, Rokos, Riad El Solh, Beirut, 1107 2050 Lebanon; 4 Helmholtz AI, Helmholtz Munich, Ingolstädter Landstraße 1, D-85764 Neuherberg, Germany

**Keywords:** Pomegranate, Machine Learning, Technological Maturity, Phenolic Maturity, Nutrition, Computational science

## Abstract

The harvesting time of pomegranates is crucial for maximizing their health benefits and market value. However, traditional methods often fail to consider the intricate interactions between environmental conditions and fruit maturity. This study is the first of its kind in Lebanon to address this limitation by applying advanced machine learning techniques to predict key food quality indicators, which can aid in forecasting or determining the optimal harvesting date. The focus is on technological and phenolic maturity. Over three months, 548 pomegranates were meticulously harvested from three distinct geographic regions in Lebanon: Hasbaya, El Jahliye, and Rachiine. By integrating environmental, physical, and geographical data, we developed predictive models, including Linear Regression (LR) and Multi-Layer Perceptron (MLP) Regressor, to estimate key food quality indicators such as Total Soluble Solids (TSS), Titratable Acidity (TA), Maturity Index (MI), phenolic content, and Color Intensity (CI). Our results demonstrated that the MLP regressor achieved high predictive accuracy, with an R-squared value of 0.84 for TA, making it a reliable tool for predicting acidity levels. The model also showed strong performance in predicting phenolic content and color intensity, with R-squared values of 0.70 and 0.65 respectively, and an average classification accuracy of 71% for categorizing polyphenol levels. Principal Component Analysis (PCA) revealed significant geographic variation in phenolic content. In El Jahliye, phenolic levels ranged from low (<185 mg Gallic Acid Equivalent (GAE) per yield of juice) to moderate (185-400 mg GAE/yield of juice). In Rachiine, levels ranged from moderate to high (>400 mg GAE/yield of juice), while Hasbaya displayed all three phenolic content levels. These findings underscore the importance of region-specific harvesting strategies. As the first study in Lebanon to utilize machine learning for predicting food quality indicators in pomegranates, it provides a novel, data-driven approach to linking these indicators with optimal harvest timing. By accurately forecasting maturity-related metrics using simple physical, geographical, and environmental features, this study offers significant implications for refining agricultural practices in Lebanon and other similar agro-ecological regions, enhancing product quality and market value.

## Introduction

Pomegranate (*Punica granatum* L.), a fruit deeply rooted in the cultural heritage of the Middle East, is celebrated for its historical importance and exceptional health benefits. Rich in polyphenols, pomegranates offer potent antioxidant, anti-inflammatory, and anticancer properties that contribute to the prevention of various chronic diseases^1^. These health-promoting attributes have led to the increasing popularity of the fruit among health-conscious consumers worldwide. In Lebanon, pomegranate cultivation extends across diverse agroecological zones, from coastal regions to higher altitudes, producing fruit with distinct characteristics influenced by the local climate and terrain^2^. Pomegranates are grown mostly for their fresh fruit, but they are also cultivated for their distinctive molasses, which is made from cultivars with a high acid content and are popular in Middle Eastern and Lebanese cuisine^2^. In Lebanon, this fruit has traditionally been regarded as a secondary crop^2^. However, despite its cultural and economic importance, the Lebanese pomegranate industry struggles to compete with imported varieties that often surpass local produce in quality.

The quality of pomegranates is intricately linked to their maturity at harvest time. Technological maturity, defined by Degree Brix, titratable acidity (TA), and firmness, plays a crucial role in determining the optimal harvest time to ensure superior fruit quality^3^. A high sugar-to-acid ratio, low acidity, and maximal sugar deposits in the pulp serve as key indicators of technological maturity^4^. The maturity index (MI) is a standard measure of ripeness in several fruits. The most frequently used MI for pomegranate is the $^{\circ }$Brix to TA ratio, often known as the sugar-to-acid ratio^5^. This stage of maturity is particularly influenced by environmental conditions, including temperature and humidity, which are crucial for proper pomegranate ripening. Additionally, the tree’s geographic distribution is affected by its susceptibility to cold temperatures^6^. Accurate determination of technological maturity is essential for maximizing the fruit’s quality and marketability.

In addition to technological maturity, phenolic maturity-characterized by the total phenolic content -is equally important. These phenolic compounds play a key role in determining a fruit’s health benefits^7,8^, its taste^9,10^, and its visual appeal, along with its color intensity closely tied to polyphenol content^11^, that make up a major part of the phenolic content^12^. Therefore, these features render phenolic maturity a critical factor in assessing overall fruit quality and are associated with the pomegranate option of selecting fruit ripening and appropriateness^13^. Among total phenols, polyphenol compounds, including anthocyanins and tannins, significantly contribute to the fruit’s color, especially the red color of arils^14^. Studies have shown that anthocyanin levels increase with pomegranate maturation, enhancing its color intensity^15^. Unlike technological maturity, which mainly concerns the pulp, phenolic maturity offers insights into the ripeness of the skin and seeds, which are rich in these health-beneficial compounds^16^. Assessing phenolic maturity in pomegranate cultivars is essential for growers to optimize the harvesting time and select premium varieties that satisfy consumer preferences and market demand, as variations in phenolic content among pomegranate varieties impact both features^17^. Recent advancements enabled phenolic quantification using UV- NIR spectroscopy, providing rapid, non-destructive, and reliable analytical^18^. This evaluation enhances the fruit’s nutritional and sensory qualities, thereby increasing its marketability.

Both maturities are crucial for determining the overall quality and marketability of pomegranates. Thus, identifying an optimal harvesting date that balances technological and phenolic maturity is essential to ensure that pomegranates reach consumers at their peak quality. This dual focus not only enhances the fruit’s sensory appeal but also maximizes its health benefits, making it imperative for producers to consider both maturity types in their harvesting strategies^19^. Climatic conditions play a pivotal role in influencing both technological and phenolic maturity. Factors such as temperature, solar radiation, humidity, and precipitation impact the development and ripening of pomegranates^15,20,21^. For instance, higher temperatures can accelerate sugar accumulation in the fruit, leading to an increase in TSS^22^, while fluctuations in humidity levels can affect acidity^23^. Additionally, the levels of antioxidants, total phenolics, and total anthocyanins in aril juice were found to be higher in pomegranates grown in regions with higher humidity, lower solar radiation, and lower temperatures^15^. These climatic factors must be carefully monitored and managed to optimize harvest timing and ensure the highest fruit quality.

Different methods have been used to identify the developmental stages of pomegranates; meanwhile, many of these processes are laborious, destructive, and unsuitable for grading and sorting. Switching to modern, non-invasive mobile and computer-based technologies can help identify the optimal pomegranate ripening phase^3^. The concept of incorporating AI technology was driven by its global advancements across various fields, including the food industry. Numerous scientific studies have demonstrated that AI is a promising alternative in this field that can replace routine methods without destroying the food matrix and can be applied to several applications. Focusing on maturity assessment and machine learning, in 2020, Karydas et al.^24^ developed a model based on machine learning for the estimation of antioxidant content in cherry fruits from images acquired from drones. In a recent study conducted in 2021, Beltrame et al.^25^ utilized digital images and independent component analysis to assess bioactive compounds in grape juice. These studies highlight the promising role of AI technology in the food industry.

Recognizing the critical need for precise maturity assessment, this study presents a novel approach by applying machine learning techniques to predict key food quality indicators, which can assist in determining the optimal harvest time for Lebanese pomegranates. For the first time, advanced machine learning models such as Linear Regression and Multi-Layer Perceptron (MLP) are employed to analyze a comprehensive dataset comprising environmental conditions, physical characteristics, and geographical features. This innovative approach represents a significant advancement for the Lebanese pomegranate industry, providing a data-driven framework to improve fruit quality, competitiveness, and consumer satisfaction. To our knowledge, no prior studies have applied machine learning models to evaluate the technological and phenolic maturity of pomegranates. This research aims to bridge this gap by combining the assessment of technological and phenolic maturity to establish reliable models for predicting critical food quality indicators, including Brix, acidity, maturity index, polyphenol content, and color intensity across various regions in Lebanon. By integrating traditional agricultural practices with cutting-edge AI technologies, this study seeks to equip local farmers with advanced tools for informed decision-making, ultimately improving the quality and market value of Lebanese pomegranates while setting a benchmark for similar applications in the region.

## Methods

### Sampling of pomegranate

Pomegranate samples were collected from three distinct locations in Lebanon: Hasbaya (a district located in Lebanon’s Nabatieh Governorate that borders Syria and is located in the southeast), El Jahliye (a village in Lebanon’s Mount Lebanon governorate’s Chouf district), and Rachiine (a village in the northern governorate of Lebanon’s Zgharta district), selected for their varying altitudes and climatic conditions. A total of 548 sweet pomegranates were harvested at different maturity stages between September and mid-November 2023. The freshly harvested pomegranates were immediately transported to the laboratory under controlled conditions to prevent any post-harvest alterations. Pomegranates are transported in temperature (around 20 $^{\circ }$C) and humidity (less than 60%). It is important to note that the transportation was done immediately (on the same day of harvesting) and the transportation period was relatively short (less than one hour). Additionally, the pomegranates are packed in suitable crates to prevent crushing and protect them from light, which could accelerate ripening and alter quality if exposed for prolonged periods. Furthermore, farmers were instructed to handle the pomegranates with care during harvesting and transport, as any damage could affect the fruit’s characteristics, as well as the imaging and measurements to be taken. Upon arrival at the laboratory, the pomegranates were assessed based on various features, including mass, volume, length, width, dilution, juice yield, location, days since harvesting, humidity, solar radiation, air temperature, and precipitation. All experimental research and field studies on pomegranate plants, including the collection of plant material, were conducted following the relevant Lebanese guidelines, regulations, and legislation. Besides, consent and approvals were obtained from the respective farmers in Hasbaya, Al Jahliyeh, and Rachiine prior to collection of the purchased pomegranates. Both R.H. and N.A. were responsible for the data collection.

### Feature parameters extraction

#### Pomegranate imaging pipeline

**Fig. 1:**
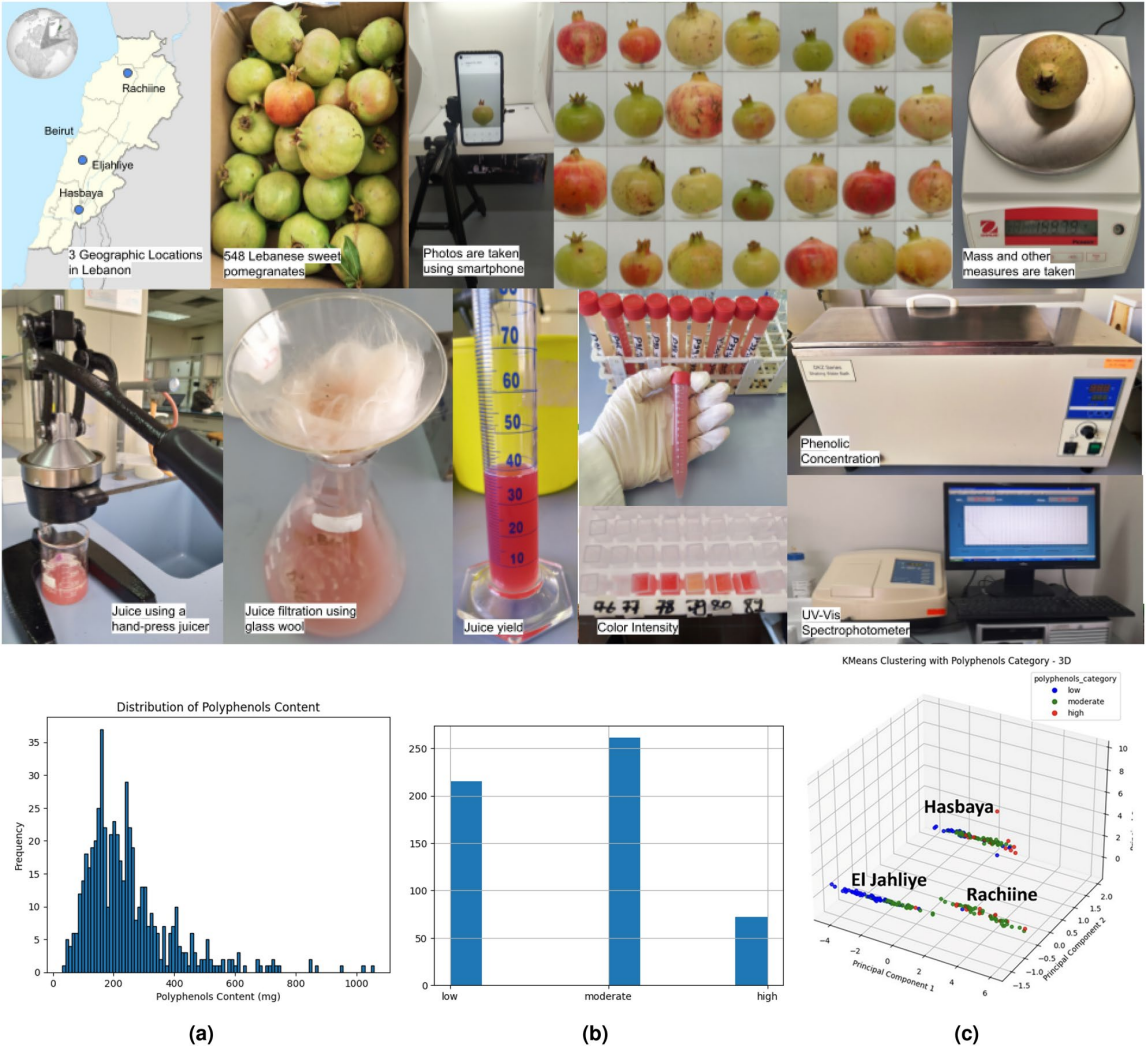
(Top) illustrates pomegranate sampling and feature parameters extractions. (Bottom) Distribution of polyphenol content (**a**), classification of pomegranates (low, moderate, and high) according to the polyphenol content (**b**), and pomegranate clustering based on polyphenol content using PCA method (**c**).

Each pomegranate was placed in a white photography box and image acquisition was performed from three different angles (*cf.* Fig.1, through a camera (48 MP, AI QUAD CAMERA) of the Redmi Note 9, Xiaomi smartphone. The camera was mounted on a tripod at a predefined fixed position, with a resolution of 2992 $\times$ 4000 px and without flash. In addition, a halved pomegranate was photographed to allow the visibility of color arils, using the same camera settings. All image data were uploaded to cloud storage to facilitate interdisciplinary collaboration across continents.

#### Physical measurements

The different characteristics of pomegranate were measured according to Fashi et al. (2020)^26^. The mass (g) of pomegranates was measured using a calibrated electronic balance (OHAUS), the volume (mL) was determined using the water displacement method, and the length (L) and width (W) (mm) were measured by a calibrated digital caliper (ingco, China). All data were recorded in an Excel sheet and uploaded to cloud storage.

#### Pomegranate juice sample preparation

Each pomegranate was halved using a sterilized sharp knife. The juice was extracted by pressing each half six times using a hand-press juicer (manual juicer SK1961211, Germany). The extracted juice was then collected and filtered through glass wool to remove any solid particles from the press cake or seeds. Finally, the juice yield from each pomegranate was measured using a graduated cylinder (mL).

### Technological and phenolic maturity measurements

#### Technological maturity measurements

Technological maturity was determined by measuring titratable acidity (TA), total soluble solids (TSS), and the maturity index (MI). Titratable acidity was determined by diluting 5 mL of pomegranate juice to 50 mL with distilled water and titrating with 0.1 mol/L NaOH, using phenolphthalein as an indicator color. The results were expressed as a percentage of citric acid^27^. For each juice sample, measurements were conducted three times, with the average values reported. The total soluble solids, indicating sugar content, were measured using a digital refractometer (Sinotech, China). Before each measurement period, the refractometer was calibrated with distilled water at 20$^{\circ }$C. The maturity index was calculated by dividing the Degree Brix by TA, providing an integrated measure of sweetness and acidity^28^.

#### Determination of phenolic content using Folin-Ciocalteu

The total polyphenol concentration and polyphenol content of pomegranate juice were quantified using the Folin-Ciocalteu method^29^. Gallic acid (3,4,5- trihydroxybenzoic acid) was used as a standard for the calibration curve (R² > 0.99). While polyphenol concentration is expressed as mg gallic acid equivalent per mL of pomegranate juice (mg GAE/mL), polyphenol content was determined by multiplying the calculated polyphenol concentration by the juice yield of each pomegranate and expressed as mg gallic acid equivalent per yield of juice (mg GAE/yield of juice). In brief, the juices were diluted 1:100, then 0.2 mL of this juice was mixed with 1 mL of 10 times diluted Folin-Ciocalteu reagent and 0.8 mL of 7.5% (w/v) sodium carbonate solution ($\hbox {Na}_2$ $\hbox {CO}_3$). All these reagents were obtained from Sigma Aldrich (Steinheim, Germany). After incubating the mixture in a water bath at $60^{\circ }$C for 10 minutes, the absorbance was measured at 750 nm using a UV-Vis spectrophotometer (OPTIMA SP-300, Kanagawa, Japan). Each sample was tested in triplicate, and the average absorbance was calculated.

#### Determination of color intensity

To determine the color intensity value, the juice was diluted to a 1/10 ratio by mixing 0.2 mL of the juice with 1.8 mL of distilled water, while the blank consisted of 2 mL of distilled water. Then, the absorbance of the color intensity was measured at 420 nm (yellow), 520 nm (red), and 620 nm (blue) using a UV-Vis spectrophotometer (SpectrumLab, Gold S54 T) connected to a computer. The sum of these three values was recorded as the color intensity^30,31^.

### Environmental conditions collection

To monitor the correlation between environmental conditions and the technological and phenolic maturity of pomegranates, environmental data for the three regions (Hasbaya, El Jahliye, and Rachiine) were collected from the Lebanese Agricultural Research Institute (LARI). The list of climatic conditions includes HC air temperature ($^{\circ }$C), solar radiation Dgt (W/m2), HC relative humidity (%), and precipitation (mm) from the beginning of September until the end of November 2023.

### Statistical analysis

The data obtained from this study were analyzed using SPSS software after being coded with an Excel program. The mean value ± standard error of the mean (SEM) value was used to present the results. *p*-values < 0.05 were considered statistically significant, indicating a confidence level greater than 95%. Statistical analysis of the data involved one-way Analysis of Variance (ANOVA) using IBM SPSS Statistics for Windows, Version 25.0 (Released 2017. IBM Corp., New York, USA).

### Machine learning methodology and predicted outcomes

The machine learning methodology aimed to predict pomegranates’ technological and phenolic maturity. The dataset incorporated physical features (mass, length, width, volume, yield of juice), geographical location, and environmental features (temperature, humidity, solar radiation, and precipitation) to predict the key food quality indicators, e.g, TA, TSS, MI, total phenolic content, and color intensity.

Machine learning models applied included the Linear Regression and Multi-Layer Perceptron (MLP) regressor for predicting continuous outcomes such as TSS, TA, and phenolic content. Logistic regression and MLP classification were used to determine maturity levels (e.g., immature, mature) for classification tasks. The predicted outcomes from these models included continuous and categorical values for TSS, TA, and phenolic content, which were crucial for determining the optimal harvest time, and the classification of pomegranates into distinct maturity stages. We opted for Linear Regression, Logistic Regression, and MLP models over complex deep learning architectures like CNNs due to the limited dataset size ($\approx$ 600 images), which increases the risk of overfitting. Since most predictive features were structured tabular data (e.g., physical and environmental attributes), traditional machine learning models were more suitable, interpretable, and computationally efficient.

In our implementation, all models utilized 10-fold cross-validation, dividing the data into 10 groups. Each fold comprised 90% training data and 10% testing data. This approach ensured that the model was thoroughly evaluated on different subsets of the data, helping to mitigate overfitting and providing more reliable performance metrics. For preprocessing, numerical features were standardized using the StandardScaler from scikit-learn^32^, while categorical features (e.g., location) were one-hot encoded. The Linear Regression model^33^ used an ordinary least squares (OLS) estimator with L2 regularization (Ridge regression) to prevent overfitting. The Logistic Regression model^33^ was trained with the ‘lbfgs’ solver and L2 penalty. For MLP models^33^, the regressor and classifier architectures included two hidden layers with (500, 1000) neurons, a ReLU activation function, and an Adam optimizer with an initial learning rate of 0.001. Training was performed for a maximum of 500 iterations, with an early stopping criterion set to halt training if no improvement was observed over 10 consecutive iterations. In our implementation, we used an 80-20 split for training and testing, respectively. This means that 80% of the data was used for training the model, while the remaining 20% was reserved for testing. By holding out a portion of the data for testing, we can evaluate the model’s performance on unseen data, providing insights into its generalization capabilities. This approach allows us to assess the model’s ability to make accurate predictions on new, unseen data, helping to ensure its reliability in real-world applications.

The models’ performance was assessed using metrics such as Mean Absolute Error (MAE) and R-squared (R^2^) for regression models, and accuracy, precision, recall, and F1-score for classification models. The final predictions enabled more precise decision-making regarding the timing of pomegranate harvesting in Lebanon, tailored to both technological and phenolic maturity.

## Results

### Kinetics of technological maturity

**Fig. 2:**
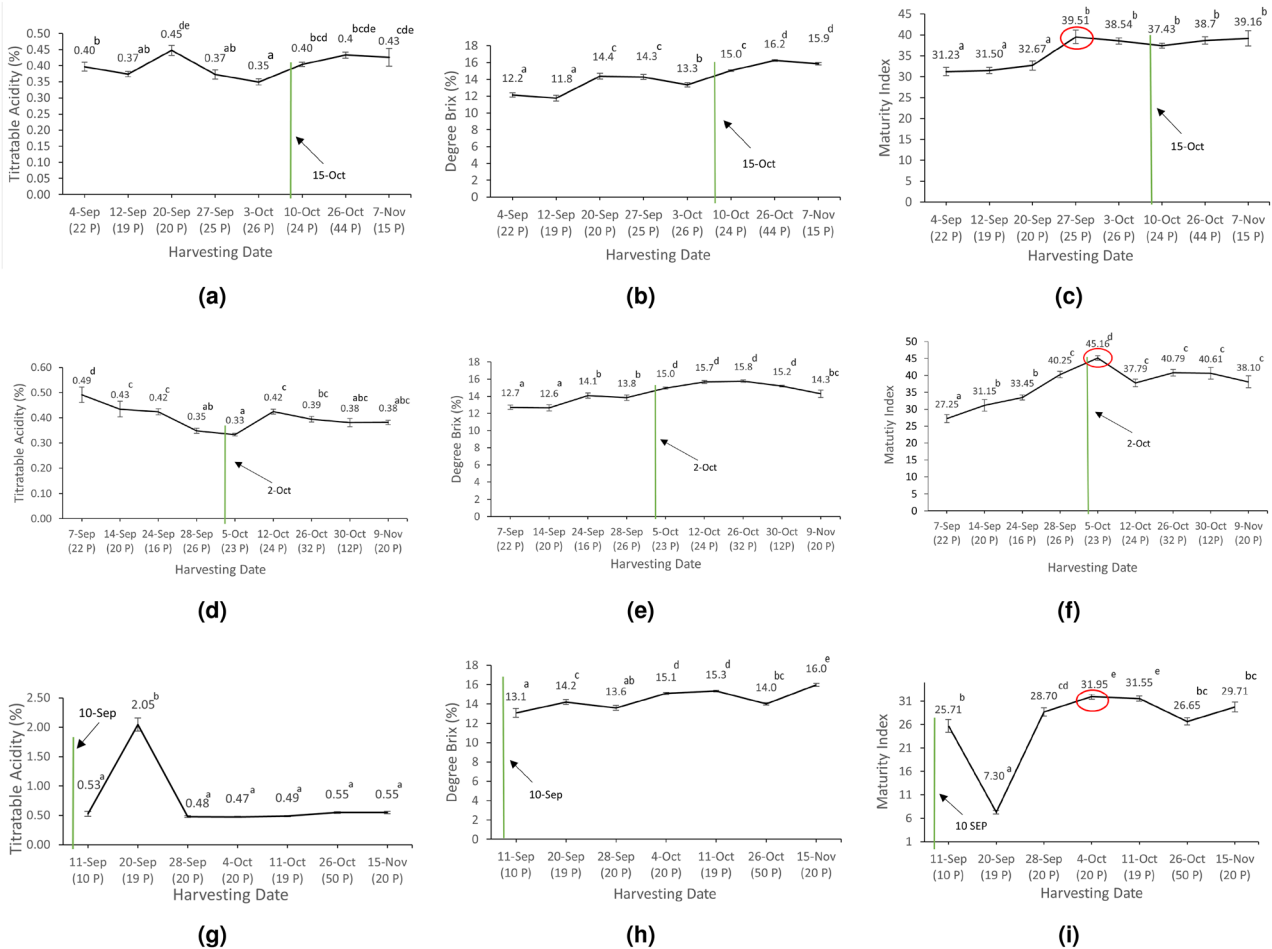
Seasonal variations. Left to Right: Titratable Acidity (%), Brix (%), and the Maturity Index (MI) of Pomegranates from Hasbaya (**a**-**c**), El Jahliye (d-f), and Rachiine (**g**-**i**) (Top to Bottom, respectively). The vertical green line represents the actual harvesting dates (collected from the farmers). The red circle corresponds to the best date to harvest pomegranate. Each mean value is labeled with superscript letters above its mark. Different letters indicate statistically significant differences between means (*p*<0.05). Numbers in parentheses indicate the number of pomegranates received on that date.

The analysis of technological maturity involved comparing the actual harvesting dates with the technological harvesting dates. Figure 2 illustrates the kinetics of technological maturity across the three sampled regions: Hasbaya, El Jahliye, and Rachiine. The maturity indices (MI) were plotted against the harvesting dates, highlighting the variance between the actual and predicted optimal technological harvesting dates.

#### Titratable Acidity (TA) variations across regions

The Titratable Acidity (TA) of pomegranates was measured during the harvest period in three distinct regions: Hasbaya (Figure 2a), El Jahliye (Figure 2d), and Rachiine (Figure 2g). In Hasbaya, TA exhibited consistent fluctuations around an average of 0.4%, with a notable decrease to 0.35% on October 3rd, which was significantly lower than other dates (*p* < 0.05) (Figure 2a). In El Jahliye, TA started at 0.49% on September 7th and gradually decreased to a minimum of 0.33% by October 5th, marking a significant difference, followed by a slight increase to 0.42% on October 12th before stabilization (Figure 2d). In Rachiine, TA initially increased from 0.53% on September 11th to peak at 2% on September 20th which was identified as a significant difference (*p*< 0.05) then decreased and stabilized around 0.48% by September 28th. The TA levels remained consistent, with no significant differences observed thereafter (Figure 2g). These trends are illustrated in Figure 2.

#### Degree Brix over the technological maturity period

Degree Brix, a measure of sugar content, increased steadily in pomegranates as they matured across the three regions Hasbaya, El Jahliye, and Rachiine (*cf.* Figure 2). In Hasbaya, Degree Brix increased gradually, starting at 12.2% on September 4th and reaching a maximum of 16.2% on October 26th. A significant difference (*p *< 0.05) was observed between pomegranates harvested at the late-ripening period (October 26) and those harvested in the earlier period (before October 26), indicating sugar accumulation throughout the maturity period (Figure 2b). The Degree Brix of El Jahliye showed a gradual increase starting at 12.7% by September 7th and peaking at 15.8% on October 26th. Subsequently, a slight decline was observed reaching 14.3% by November 9th which represents a significant difference (*p* < 0.05) (Figure 2e). For Rachiine, the Brix levels demonstrated a consistent increase from 13.1% on September 11th to a maximum of 16% on November 15th, indicating a significant difference (*p *< 0.05) from the other dates, with minor fluctuations between these two dates. The seasonal variations in Degree Brix are presented in Figure 2.

#### Monitoring the Maturity Index (MI)

The Maturity Index (MI), which influences the taste of pomegranate juice, showed significant increases in the three regions (*cf.* Figure 2). In Hasbaya, MI increased significantly (*p* < 0.05) from 31.23 on September 4th to 39.51 on September 27th then fluctuated slightly, without significant differences for the other dates. This result indicates that the technological maturity harvesting date should be October 26th where the Degree Brix is significantly high and the TA and MI values stable with no significant difference. However, farmers started harvesting on October 15th. For El Jahliye, MI levels showed a significant increase (*p* < 0.05) from 27.25 on September 7th to 45.16 on October 5th. Subsequently, MI levels decreased slightly to 37.79 showing no significant differences from October 12 till November 9. This suggests that harvesting of pomegranates should ideally start by October 5th; however, farmers began harvesting on October 2nd, which is still considered acceptable. For Rachiine, there was a notable and significant decrease in MI dropping from 25.71 on September 11th to 7.30 on September 20th. Subsequently, MI values increased, reaching a maximum of 31.95 by October 4th. The MI then decreased to 26.65 by October 26th but rose again without significant difference. This indicates that the optimal harvesting date should be October 4th, however, farmers start harvesting by September 10th which is considered too early, as shown in Figure 2. Farmers in the three regions began harvesting based on the visual assessment of the pomegranates such as the red color, larger size, and taste of the pomegranates.

### Kinetics of polyphenols production and color intensity evaluation

**Fig. 3:**
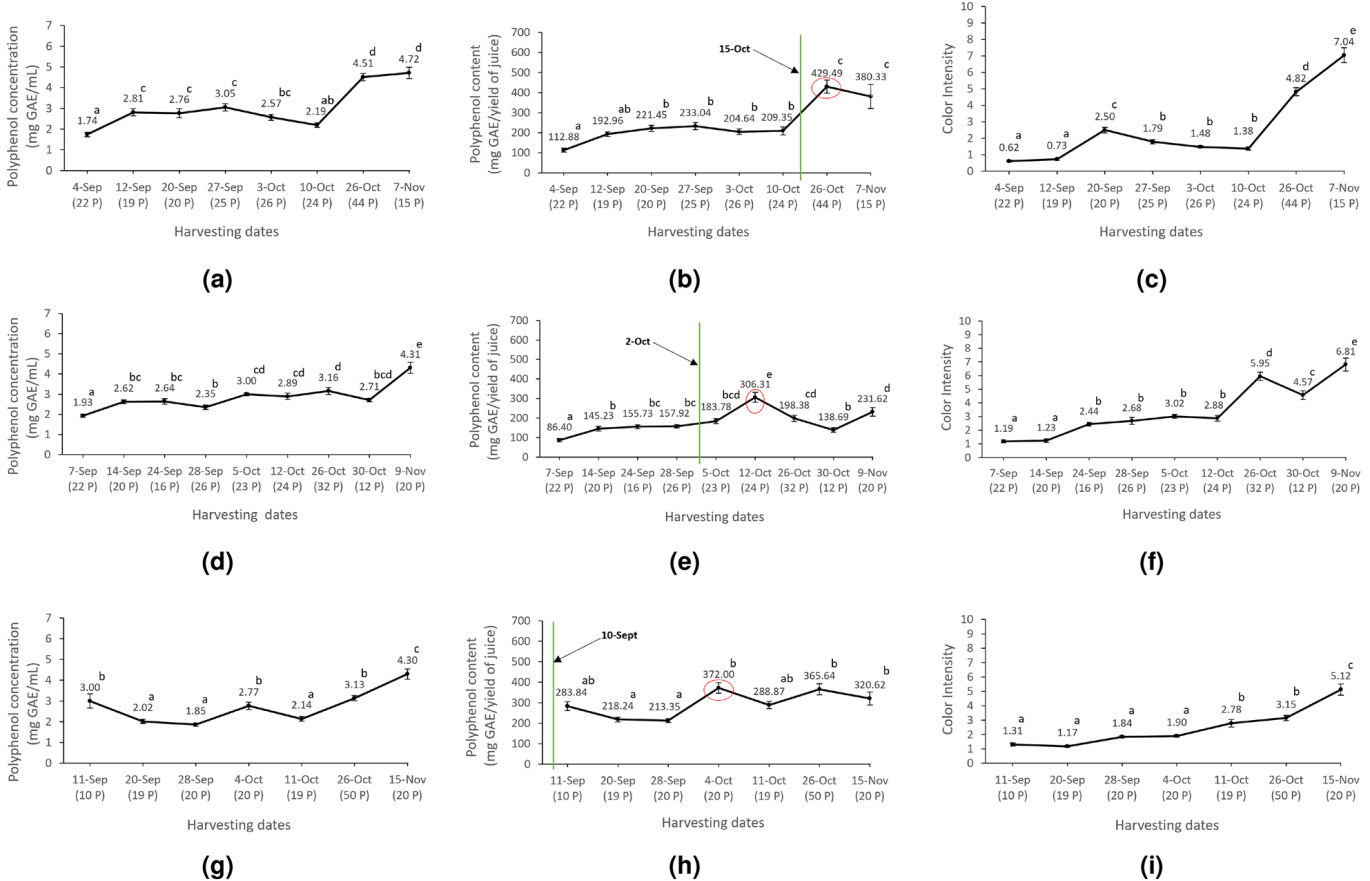
Assessment of Polyphenols concentration, content, and color intensity of 548 pomegranates in three geographic locations during different harvesting dates over three months of 2023 (September, October, and November). From Left to right: Polyphenol concentration (mg GAE/ml), Polyphenol content (mg GAE/yield of juice), and color intensity. From Top to bottom; Hasbaya (**a**-**c**), El Jahliye (**d**-**f**), and Rachiine (**g**-**i**), accordingly. The vertical green line represents the actual harvesting dates (collected from the farmers). The red circle corresponds to the best date to harvest pomegranates. Each mean value is labeled with a letter above its mark. Different letters indicate statistically significant differences between means (*p*<0.05). Numbers in parentheses indicate the number of pomegranates received on that date.

Polyphenol concentration was assessed to evaluate polyphenol kinetics over the harvesting dates. The results illustrated in Figure 3 revealed a significant increase with distinct regional differences. Indeed, in Hasbaya (Figure 3a), the average polyphenol concentration increased from 1.74 mg GAE/mL on September 4th to 4.51 mg GAE/mL on October 26th, with no significant difference from 4.72 mg GAE/mL on November 7th. For El Jahliye, polyphenol concentration increased significantly from 1.93 mg GAE/mL on September 7th to 4.31 mg GAE/mL on November 9th (Figure 3d). For Rachiine, the polyphenol concentration increased from 3.0 mg GAE/mL on September 11th to 4.3 mg GAE/mL on November 15th (Figure 3g).

The analysis of phenolic maturity involved comparing the actual harvesting dates with the proposed optimal harvesting dates based on polyphenol content values. These values have been chosen to provide a more accurate assessment considering the yield of juice. Figure 3 illustrates polyphenol content across the three sampled locations: Hasbaya, El Jahliye, and Rachiine. The polyphenol content average in Hasbaya increased from 112.88 mg GAE/yield of juice on September 4th to 429.49 mg GAE/yield of juice on October 26th, then it showed a non-significant decreasing on November 7th (Figure 3b). This result may indicate that the phenolic maturity harvesting date should be October 26th, where the polyphenol content is significantly high. However, farmers started harvesting by October 15th. El Jahliye region demonstrated a significant variation of the polyphenol content average over the harvesting dates with a peak of 306.31 mg GAE/yield of juice on October 12th (Figure 3e). This finding suggests that harvesting of pomegranates may start by October 12th. However, farmers began harvesting on October 2nd. In Rachiine region, the polyphenol content average (Figure 3h) started with a value of 283.84 mg GAE/yield of juice on September 11th to reach a peak on October 4th, where the average reached 372.0 mg GAE/yield of juice, followed by fluctuations of increases and decreases observed between 11th October and 15th November, with no significant difference. This observation suggests that the phenolic maturity harvesting date may be October 4, when the polyphenol content is significantly high. However, farmers started harvesting by September 10th. Overall, these results demonstrate a general increase of polyphenol content over time to reach a peak at a defined harvesting date and that Hasbaya exhibited the highest polyphenol content, reaching up to 429.49 mg GAE/yield of juice, followed by Rachiine and El Jahliye, where the content stabilized around 372.0 mg GAE/yield of juice and 306.31 mg GAE/yield of juice, respectively.

The evaluation of color intensity across these regions and over the harvesting dates showed a strong increase in color intensity over the harvesting time, and a correlation between polyphenol concentration and color intensity, Figure 3. As polyphenol levels increased, pomegranate juice color intensity increased. By the 7^th^ of November, Hasbaya’s pomegranates, which had the highest polyphenol concentration, also displayed the most intense color, with a color intensity value of 7.04 (Figure 3c). El Jahliye and Rachiine followed similar trends, with lower color intensities of 6.81 and 5.12, respectively (Figure 3f, i).

#### Distribution and classification of polyphenol content in pomegranate juice per location

To explore polyphenol level distribution in the 548 pomegranate samples, pomegranate frequency has been calculated for different polyphenol content values (Figure 1a). For instance, it was demonstrated that pomegranate frequency varies across different polyphenol levels (Figure 1a). Following this analysis, polyphenol values have been classified as low (polyphenol content less than 185 mg GAE/yield of juice), moderate (values ranging between 185 and 400 mg GAE/yield of juice), and high (values above 400 mg GAE/yield of juice). According to Figure 1b, the moderate category exhibited a high number of pomegranates. Following this was the low category, which had fewer pomegranate number. Notably, the high polyphenol content category showed a lower count of pomegranates. To check variation in polyphenol content categories across the three regions, a PCA method has been elaborated and the results are illustrated in Figure 1c. PCA analysis shows that pomegranates harvested from Hasbaya represented the highest level of polyphenol, followed by Rachiine, then El Jahliye, which represented the lower polyphenol content. While Hasbaya showed the three levels of polyphenol content, Rachiine exhibited moderate and high levels of polyphenol content. However, for El Jahliye, this content ranged between low and moderate levels. Figure 1c shows also that at the beginning of the pomegranate collection, the quantity of polyphenols was low, but over the harvesting time, this level increased. Overall, these results demonstrate that polyphenol content varies with pomegranate maturity and across geographic locations.

### Correlation between technological and phenolic maturity

Table 1 presents a comparison of actual harvesting dates with the proposed technological and phenolic maturity dates for pomegranates in Hasbaya, El Jahliye, and Rachiine. By comparing the optimal (recommended) harvesting dates of phenolic and technological maturity data, it is found that Hasbaya farmers could start harvesting by October 26th which demonstrated the highest brix and MI (although not statistically significant) aligned with the best phenolic maturity. Therefore, 26th of October was the proposed date to start picking pomegranates to optimize the quality of pomegranates. However, farmers started harvesting by October 15th which is quite early. As for El Jahliye, it is recommended to start collecting pomegranates between October 5th and October 12th to ensure optimal pomegranate quality and health benefits. This is referred to the analysis of MI, polyphenol content, and color intensity of pomegranates which indicates the highest MI on October 5th while the peak levels of polyphenol content and color intensity were on October 12th. Surprisingly, El Jahliye farmers began picking on October 2nd which is near the optimal harvesting date range. For Rachiine, the analysis of both the technological and phenolic maturity suggests that October 4th represents the optimal harvesting date. On this particular date, MI and polyphenol content along with the color intensity of pomegranates reach their peak. Nonetheless, the actual harvesting date by farmers was September 10th, which is considered earlier than the recommended date which could have an impact on the quality of pomegranates.

**Table 1:** Comparison of Actual, Technological, Phenolic, and Recommended Harvesting Dates of Pomegranates.

**Region**	**Actual Harvesting Date**	**Technological Harvesting Date**	**Phenolic Harvesting Date**	**Recommended Harvesting Date**
Hasbaya	October 15th	October 26th	October 26th	October 26th
El Jahliye	October 2nd	October 5th	October 12th	October 5th - 12th
Rachiine	September 10th	October 4th	October 4th	October 4th

### Impact of environmental conditions on technological maturity and polyphenol content

**Fig. 4:**
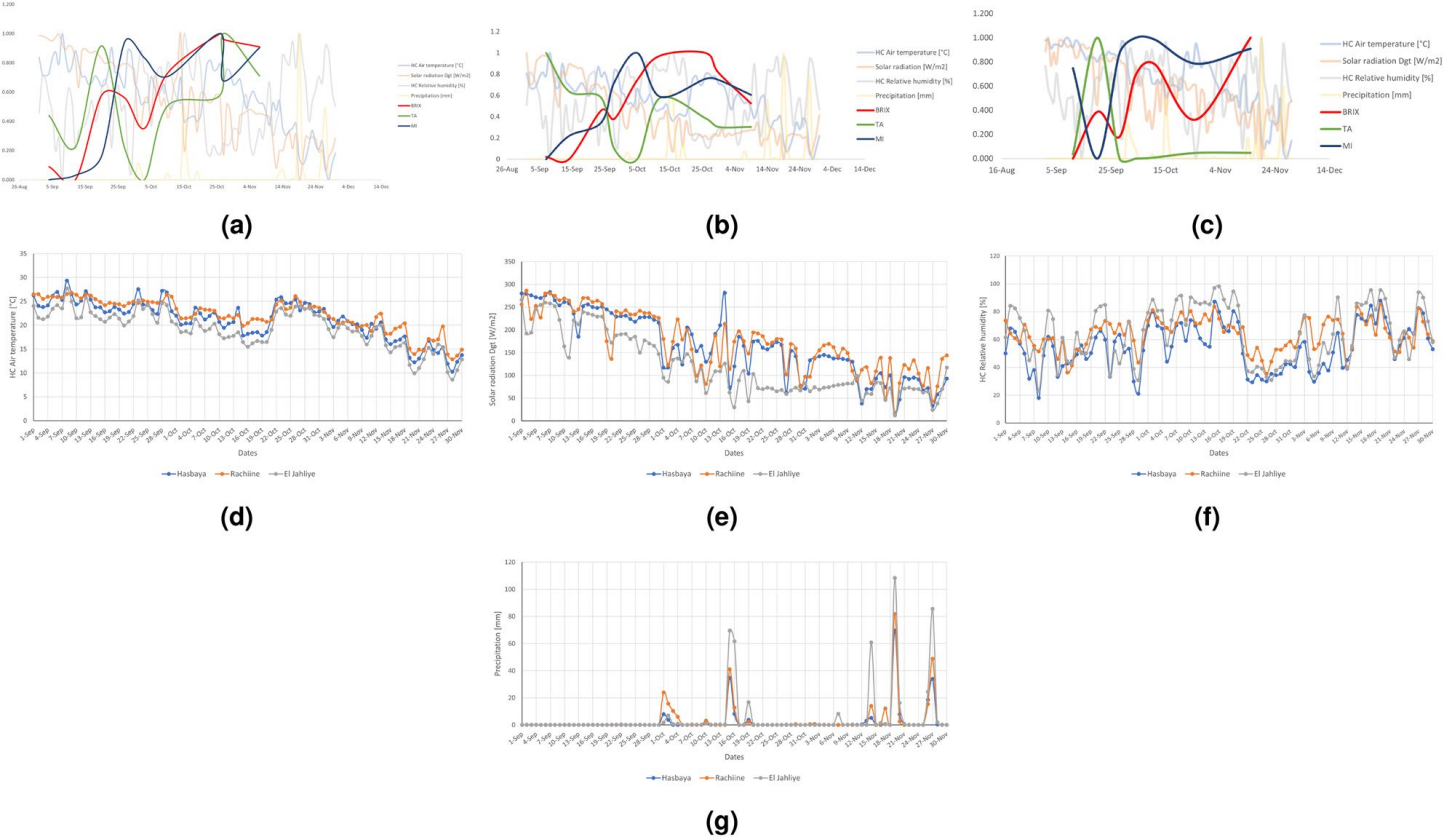
Correlation of Brix, TA, and MI with the climatic Factors (Temperature, Solar radiation, Humidity, and Precipitations) in Hasbaya (**a**), El Jahliye (**b**), and Rachiine (**c**) during the harvesting dates and the impact of environmental conditions on polyphenols content across Hasbaya, Rachiine and El Jahliye in Lebanon during harvesting dates in September, October, and November 2023. d: Changes in Air temperature. e: Changes in solar radiation. f: Changes in humidity. g: Changes in precipitation.

Climatic conditions, including temperature, humidity, solar radiation, and precipitation, play a critical role in influencing technological and phenolic maturity. In this section, we investigated the impact of environmental conditions (temperature, humidity, solar radiation, and precipitation) on the technological and phenolic maturity of pomegranate. While comparing the climatic conditions to the technological maturity features of Hasbaya, it is suggested that environmental factors, such as increased precipitation, reduced solar radiation, and lower air temperatures, could influence the trends in titratable acidity (TA) and the maturity index (MI). For example, during Winter, cooler periods between October 2nd and 20th, TA tended to increase, while MI showed a slight decline, and Degree Brix appeared less sensitive to these variations, this analysis implies that precipitation and air temperature fluctuations could play a role in affecting the trends of TA and MI, alongside with other possible influences (Figure 4a). In El Jahliye, between September 5th and October 2nd, environmental factors such as elevated air temperatures and solar radiation, along with low humidity, may have contributed to an increase in Degree Brix and maturity index and a decrease in TA trend. After rainfall began on October 2nd, minor fluctuations were observed in all parameters, possibly due to the reduction in solar radiation and air temperatures. These changes could be related to the environmental conditions, though other factors might also have contributed to the observed patterns in the maturity indicators. (Figure 4b). In Rachiine, during the first week, high stable air temperatures and solar radiation, low humidity, and the absence of precipitation coincided with an increase in TA and a decline in MI, which may suggest that these trends are not affected by climatic conditions. Other factors may be responsible for this shift. Following this, a marked decrease in TA was observed alongside increases in both Brix and MI. From October 1 st, the onset of rainfall, increased humidity, reduced temperature, and solar radiation coincided with a stabilization in TA and a modest decline in Brix and MI, suggesting that these environmental shifts may be associated with changes in these maturity indicators. However, the possibility that other, non-climatic, factors contribute to these trends cannot be excluded. (Figure 4c). Regarding the phenolic maturity, the comparison depicted in Figure 4 and Figure 4c underscores that El Jahliye exhibits a notably lower polyphenol content in comparison to Hasbaya and Rachiine. Upon examining the climatic conditions across these regions, it becomes evident that El Jahliye experiences lower levels of temperature and solar radiation (Figure 4d,e). However, it is noteworthy that this region boasts higher levels of humidity and precipitation (Figure 4f,g). This correlation suggests that the reduced polyphenol content observed in El Jahliye seems to be influenced by its higher precipitation levels and lower solar radiation and temperature values. In contrast, Hasbaya and Rachiine characterized by higher temperature and solar radiation, and less precipitation and humidity, showed a significant increase in polyphenol content level. Overall, these results show that the variation in technological maturity and polyphenol content across the Lebanese regions may be attributed to changes in climatic conditions.

### Data exploration and machine learning models

####  Regression model results

**Fig. 5:**
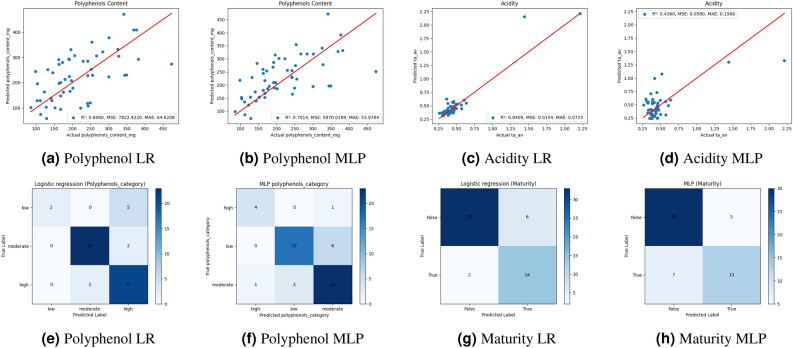
Regression Results of Polyphenols Content (**a**-**b**) and Acidity (**c**-**d**) for Logistic Regression (LR) and MLP. The confusion matrices of the Polyphenol’s category (**e**-**f**) and Maturity (**g**-**h**) for LR and MLP.

The results of applying linear regression and MLP regressor models to predict various outcomes are presented in Table 2, and Figure 5a-d. In this section, all models are validated with 10-fold cross-validation for robustness purposes. For the outcome of polyphenol content (mg GAE/yield of juice), the linear regression model achieved an R² value of 0.6060, indicating that approximately 60.60% of the variance in polyphenol content was explained by the model. The Mean Absolute Error (MAE) for this outcome was 64.6208. The linear regression model also performed reasonably for predicting color intensity, with an R² value of 0.6153 and an MAE of 0.9411. For the outcome of the Maturity Index, the linear regression model showed an R² value of 0.4320 and an MAE of 5.2872. For Degree Brix and Acidity, the linear regression model exhibited low R² values of 0.3582 and 0.4360, respectively, with corresponding MAE values of 1.0101 and 0.1568. These results provide insights into the predictive performance of the linear regression model across different target outcomes, highlighting its ability to rather capture the relationships between input features and target variables in the dataset. In contrast, the MLP regressor yielded an R² value of 0.7014 for the outcome of the polyphenol content (mg GAE/yield of juice), indicating that approximately 70.14% of the variance in the polyphenol content was explained by the model. The Mean Absolute Error (MAE) was 53.9784. The MLP regressor also performed reasonably well in predicting color intensity, with an R² value of 0.6599 and an MAE value of 0.8222. Regarding the Maturity Index, the MLP model showed a lower R² value of 0.565 and an MAE of 4.299. For Degree Brix and Acidity, the MLP regressor exhibited R² values of 0.440 and 0.841, respectively, with corresponding MAE values of 0.888 and 0.0725.

These results demonstrate the efficacy of the MLP regressor (over the linear model) in capturing the relationships between the input features and the target outcomes, providing valuable insights into the predictive performance of the model across different variables.

**Table 2:** Regression results of 10-fold Cross-Validation, reporting $R^{2}$, $MSE$ and $MAE$. Metrics in boldface demonstrate the best performance.

Model	Outcome	10-fold Cross-validation Mean (Median) ± Std		
		$R^{2} \uparrow$	$MSE \downarrow$	$MAE \downarrow$
Linear Regression	Polyphenols content (mg GAE/yield of juice)	$0.6060 (0.6145) \pm 0.0808$	$7822.4220 (8082.29) \pm 1978.7504$	$64.6208 (68.9721) \pm 7.8466$
	Color intensity	$0.6153 (0.6223) \pm 0.0575$	$1.6479 (1.5141) \pm 0.2964$	$0.9411 (0.9132) \pm 0.0692$
	Maturity index	$0.4320 (0.4598) \pm 0.0601$	$45.6875 (46.2443) \pm 8.2445$	$5.2872 (5.2289) \pm 0.5116$
	Degree brix (%)	$0.3582 (0.3373) \pm 0.0719$	$1.7772 (1.5381) \pm 0.4433$	$1.0101 (1.0094) \pm 0.0765$
	Acidity (%)	$0.4360 (0.4338) \pm 0.0658$	$0.0580 (0.0583) \pm 0.0189$	$0.1568 (0.1592) \pm 0.01$
MLP Regressor	Polyphenols content (mg GAE/yield of juice)	${\varvec{0.7014 (0.7097) \pm 0.0732}}$	${\varvec{5870.0189 (6485.6017) \pm 1527.9304}}$	$\varvec{53.9784 (58.2323) \pm 6.2308}$
	Color intensity	${\varvec{0.6599 (0.6781) \pm 0.0818}}$	${\varvec{1.4645 (1.1848) \pm 0.3424}}$	$\varvec{0.8222 (0.7484) \pm 0.0899}$
	Maturity index	${\varvec{0.5650 (0.6200) \pm 0.0888}}$	${\varvec{34.1511 (30.1061) \pm 6.9254}}$	$\varvec{4.2990 (4.1852) \pm 0.2815}$
	Degree brix (%)	${\varvec{0.4404 (0.4646) \pm 0.1226}}$	${\varvec{1.5596 (1.3766) \pm 0.5069}}$	$\varvec{0.8882 (0.8742) \pm 0.1040}$
	Acidity (%)	${\varvec{0.8409 (0.8654) \pm 0.0330}}$	${\varvec{0.0154 (0.0147) \pm 0.0055}}$	$\varvec{0.0725 (0.0720) \pm 0.0063}$

#### Classification model results

In this section, the performance of the logistic regression and MLP models was evaluated using 10-fold cross-validation for the models indicated in Table 3 and Figure 5(e, f, g, h). For both models, maturity classification, and logistic regression achieved an accuracy of 79.01%, and F1-score values of 76.70%. MLP models exhibited slightly lower robust performance, achieving an accuracy of 77.58% and an F1-score value of 76.13% for maturity category classification. Similar to maturity evaluation, these two models have been used to evaluate the polyphenol’s category, determined experimentally from the polyphenol content distribution, and classified into three levels: low (less than 185 mg GAE/yield of juice), moderate (185–400 mg GAE/yield of juice), and high (above 400 mg GAE/yield of juice). The logistic regression, Figure 5e shows that the model can predict a high (21/23: 91%) and moderate (23/25: 92%) polyphenol content more than the low level (2/7: 28%). However, the MLP model can predict the three categories in percentages corresponding to low (17/23: 73.9%), moderate (23/27: 85%), and high (4/5: 80%) (Figure 5f). The results indicate an accuracy of 73.19% for polyphenol’s category classification, with an F1-score value of 72.20%. MLP models exhibited a slightly less robust performance, achieving an accuracy of 71.19% and an F1-score of 70.87% for polyphenol category classification (Table 3).

**Table 3:** Classification results of 10-fold Cross-Validation, reporting Accuracy, Precision, Recall, and F1-score. Metrics in boldface demonstrate the best performance.

Model	Outcome	10-Fold Cross-validation Mean (Median) ± Std			
		Accuracy	Precision	Recall	F1 score
Logistic Regression	Polyphenol’s category	${\varvec{0.7319 (0.7364) \pm 0.0539}}$	${\varvec{0.7640 (0.7651) \pm 0.0677}}$	${\varvec{0.7147 (0.7087) \pm 0.0518}}$	$\varvec{0.7220 (0.7192) \pm 0.0456}$
	Maturity	${\varvec{0.7901 (0.7909) \pm 0.0420}}$	$0.7807 (0.7903) \pm 0.0526$	$0.7662 (0.7700) \pm 0.0482$	$\varvec{0.7670 (0.7762) \pm 0.0458}$
MLP Regressor	Polyphenol’s category	${0.7119 (0.7364) \pm 0.0846}$	$0.7617 (0.7669) \pm 0.0863$	$0.7042 (0.7038) \pm 0.0865$	$0.7087 (0.7185) \pm 0.0849$
	Maturity	$0.7758 (0.8000) \pm 0.0906$	${\varvec{0.7909 (0.8061) \pm 0.0711}}$	${\varvec{0.7779 (0.7884) \pm 0.0680}}$	$0.7613 (0.7907) \pm 0.0883$

## Discussion

The results of this study highlight the critical role of technological and phenolic maturities in predicting the optimal harvest time for pomegranates, particularly through the accurate modeling of key parameters such as Titratable Acidity (TA), Total soluble solids (TSS), Maturity Index (MI), phenolic content, and Color Intensity (CI). These parameters, which serve as indicators of both technological and phenolic maturity, directly influence the quality and nutritional value of the fruit, making them essential for determining the ideal harvest time.

Primarily, the technological maturity findings showed that TA is one of the contributing factors in pomegranate taste and overall quality^34^. TA for Hasbaya fluctuates over the ripening period. This trend is in concordance with the findings of Shwartz et al.^35^, who reported that the decrease in TA of the accession ‘121-22’ was not significant. In El Jahliye, TA levels decreased all over the harvesting period, which is in line with the reduction in TA observed all over the technological maturity period of pomegranates as reported in^36,37^. In Rachiine, the trend of TA increased at the beginning and then stabilized till the end of the harvest season, which is in agreement with Varasteh et al.^38^ who reported a similar trend of TA increasing initially and subsequently declining. In addition, the fact that the acidity stabilized later in the season aligns well with the study of Ben-Arie et al.^36^ who observed that TA remained constant after the second half of September.

Another factor contributing to technological maturity is the degree brix, which showed variations across the three different regions (Hasbaya, El Jahliye, and Rachiine) during the harvest period, with a notable increase in Brix levels for Rachiine and Hasbaya, ranging from a minimum of 12% to a maximum of 16%. These results are aligned with previous studies^39–41^ that reported an increase in the trend of Brix during the technological maturity period. However, the Brix levels in El Jahliye increased during the maturity period, peaking at 15.8% by October 26th to decrease and reach 14.3% by November 9th which is in agreement with Boussaa et al.^42^ who reported an increase in TSS until mid of October and then decreased once maturity was complete.

As a result of TSS and TA changes, the MI increased with the delay of the harvest. The maturity index for Hasbaya increased throughout the harvesting period, ranging from a minimum of 31 to a maximum of 39, which is in agreement with the findings of Fawole & Opara^43^, who also stated an increase in values of MI. Similarly, the maturity index of El Jahliye increased from 27.25 to a maximum of 45.16, which aligns also with the findings of Fawole & Opara^43^. However, MI for Rachiine was affected by TA at the beginning of the harvesting dates, which caused the decrease in MI and then totally continued increasing until the end of the harvesting dates.

Secondarily, the assessment of phenolic maturity for pomegranate samples collected from three different geographic locations in Lebanon showed that polyphenol concentration trends increased over the harvesting dates. This observation may be attributed to the greatest accumulation of anthocyanins in the arils during ripening. This result is in agreement with previous studies that found an increase in total phenolic in muscadine grapes^44^, in sweet cherry^45^, and in one pomegranate accession “the 101-2”^35^. However, when considering the polyphenol content, it was evident that this parameter increases at certain times, with variations between regions, and then decreases again. This may be attributed to the yield of juice that varies between the harvesting dates.

To determine the optimal harvesting date of pomegranates, three key parameters were considered: 1) the date with the highest polyphenol content to benefit from its biological value^46^ 2) the timing of harvest, ensuring it occurs before the end of the season when fruits begin to fall; and 3) the color intensity of the fruit which contributes to an attractive juice color and plays a key role in consumer appeal^47^. Additionally, juice yield was considered as a separate factor, with an emphasis on maximizing production.

Taking into consideration polyphenol content, we have predicted harvesting times for Hasbaya, El Jahliye, and Rachiine, corresponding to October 26th, October 12th, and October 4th, respectively. Then, assessing the color intensity on these specified days has shown that this parameter is not the highest, but it has a good value to attract consumers. Furthermore, if we let pomegranate fruit sit for more days to have better color intensity, this may affect fruit firmness and appearance^42^. Choosing these specific dates showed a deviation between the optimal and actual harvesting dates, suggesting more awareness campaigns for the farmers to be implemented.

The evaluation of pomegranate juice color intensity showed an increase over time in the three locations. This result may reveal a correlation between pomegranate ripening and color intensity. This finding is in line with Aglar et al.^48^who found that color intensity increased with cherry fruit ripening. Moreover, it is essential to note that the highest color intensity corresponded to the highest polyphenol concentration at the same harvesting time. That may indicate that an increase in polyphenol concentration reflects an increase in anthocyanin concentration during pomegranate ripening. This result aligns with the findings of Kulkarni & Aradhya^37^, who demonstrated that a deeper red color of pomegranate juice reflects anthocyanin content.

Following the evaluation of phenolic maturity and color intensity over ripening, we were interested in exploring the polyphenol content distribution in pomegranate samples, and the impact of geographic locations on this parameter. Distribution and classification analysis have shown three categories of polyphenol content (low, moderate, and high) and that pomegranates were not equally distributed across them. This finding may be attributed to cultivar and genome variations as shown by Montefusco et al.^17^ who reported a genotype-dependent variability in total phenolic compounds. Regarding the geographic locations, PCA analysis has demonstrated that pomegranate samples could be clustered into three different clusters based on their polyphenol content, where each cluster corresponds to a specific geographic location. This result aligns with the findings of Ghasemi-Soloklui et al.^6^ that demonstrated the influence of geographic conditions on phenolic content.

Our results reveal significant differences between recommended harvest dates based on maturity indices and the actual harvest dates used by farmers, with consequences on pomegranates quality and health benefits. In Hasbaya, the results indicated that the optimal harvest date based on our findings while comparing the phenolic and technological maturity dates is October 26th. However, farmers started by October 15th which is considered earlier than the recommended day. This early harvesting may result in pomegranates that have not attained their full flavor, juiciness, and nutritional value. For El Jahliye, our study revealed that the ideal harvest period is from October 5th to October 12th. This phase corresponds to the highest levels in MI, phenolic content, and color intensity, suggesting the greatest quality and health benefits. The real harvest by El Jahliye farmers begins on October 2nd, which is quite close to this period, indicating that with slight modification, farmers might perfectly optimize fruit quality. As for Rachiine, the ideal harvesting date is October 4th while Rachiine farmers started on September 10th which is considered too early. Farmers report that they start harvesting earlier because the pomegranates begin turning red, increasing in size, and in some cases, cracking and splitting, or even falling from the tree. These visible and physical changes are perceived as signs of ripeness; however, they do not always align with the optimal technological and phenolic maturity recommended for optimal quality. This major difference from the optimal date most likely results in a harvest of unripe pomegranates, significantly reducing their quality, flavor, and nutritional content, including lower polyphenol content, low color intensity, and low sugar content with high acidity.

It is well known that technological maturity and polyphenol levels are affected by the climatic conditions of regions^49^. In Hasbaya, when rainfall began during the ripening period, the drop in titratable acidity (TA) was slowed down, and instead titratable acidity levels began to increase. These findings are consistent with similar results by^23^ who reported that rainfall can slow TA decreases during fruit development. Furthermore, high temperatures and solar radiation, coupled with the absence of precipitation, were associated with reduced TA levels and increased Brix and maturity index (MI) trends in El Jahliye. This aligns with findings by Crespo et al.^50^, who reported a drop in TA in grapes as air temperature rose. Similarly, Dong et al.^23^ showed that optimal lighting conditions and higher temperatures support decreases in fruit acidity. While these findings suggest that temperature, solar radiation, and precipitation significantly impact acidity and sugar accumulation, observations in Rachiine indicate that even in the absence of precipitation, coupled with high temperatures, and solar radiation coincided with an increase in acidity and a decrease in the maturity index (MI). This unusual trend suggests that, while climate factors like temperature, solar radiation, and precipitation affect acidity and sugar levels, there may be other factors to consider. A study done by Ghasemi-Soloklui et al.^6^has shown that the biochemical makeup and mineral components of pomegranate fruit and arils differ depending on factors such as genetics, growing area, climate, maturation, harvesting practices, and storage conditions. This highlights the demand for more studies to fully understand the effect of climate on technological maturity, as well as the potential influence of other factors on these maturity trends. Concerning the impact of climatic conditions on the polyphenol content, it was observed that El Jahliye has the highest precipitation level, the lowest temperature, and solar radiation levels compared to Hasabaya and Rachiine. Furthermore, polyphenol content has shown lower values over the harvesting dates in El Jahliye. This result aligns with the data presented in previous studies, e.g^6,15,19^, where they showed that precipitation increasing and other climatic conditions decreasing led to a decrease in polyphenol levels. It is important to note here that previous studies have worked on climatic conditions; they considered not a couple of months like in our study but years and seasons to study the effect of climatic conditions on polyphenol levels. Nevertheless, our study confirms the impact of environmental conditions and geographic locations on polyphenol content.

AI technologies have been used in many fields, among which the food industry encompasses various goals aimed at enhancing efficiency, quality, safety, sustainability, and customer satisfaction^51^. To assess and predict technological maturity, polyphenol content, and color intensity in pomegranate juice in Lebanon, two AI models (linear regression and MLP regression) have been developed and tested, taking into consideration 13 features. While assessing the predictive performance of the different regression models on Titratable acidity, Degree Brix, and Maturity Index of the pomegranate samples, it was observed that the MLP and the Linear regression models differ significantly in their prediction abilities, where the MLP model which is considered more complex than the linear regression model showed high capabilities of predicting the acidity with a high R² value of 0.841 and a low MAE of 0.0725 which means that the model predictions are close to the actual data, indicating the effectiveness of the model in capturing acidity while the linear regression model showed a contrast results with a low R² of 0.4360 and an MAE of 0.1568 which failed to accurately predict the acidity. For Degree Brix, both the MLP Regressor and the linear regression models showed similar performance in terms of R² equal to 0.440 and 0.3582 respectively, and corresponding to MAE levels equal to 1.0101 and 0.8882 respectively. These results reflect some limitations in their ability to capture the Brix degrees. Moreover, the prediction of the maturity index by both models remains challenging with the low R² (0.565 and 0.4320). Regarding polyphenol content and color intensity outcomes, the MLP regressor showed the best R² (0.7014 and 0.6599) and MAE values. Comparing these two outcomes, we can conclude that the best prediction is the polyphenol content because R² is the closest thing to 1, but for the color intensity, the R² is moderate and acceptable, but can be improved with more samples of pomegranate. For the assessment of the logistic regression and the MLP models using 10-fold cross-validation and confusion matrices present marked performance differences. The logistic regression model showed a high accuracy percentage for maturity of 79.01% with an F1 score of 76.70% compared to the MLP model which presents an accuracy percentage of 77.58% and an F1 Score of 76.13%. The logistic regression shows low rates of false negatives and false positives, which demonstrate the sensitivity and specificity of the logistic model. However, the MLP model presents a high number of false negatives demonstrating the model’s struggle with recognizing positive data. These results indicate that the logistic model is considered a more reliable model to predict maturity. Applying these two models, to polyphenol categories (low, moderate, and high), we note that the first model predicts moderate and high levels better with an accuracy of 0.7319, while the second model can predict all the polyphenol levels with an accuracy of 0.7119. This means that logistic regression can correctly predict around 73% of tested pomegranates, and MLP regressors can only correctly predict around 71%. From these results, and for the first time in Lebanon, the AI model has proven to be effective for technological maturity and polyphenol content classification and prediction.

### Conclusions and future work

This study is considered the first of its kind in Lebanon to assess both technological and phenolic maturity in pomegranate from three distinct geographic regions-Hasbaya, Rachiine, and El Jahliye-while also incorporating AI technologies for predicting key food quality indicators. Our results demonstrate that acidity levels decline, while Brix and maturity index increase over the harvest period, with small deviations that could be related to the regions and climatic conditions. Additionally, our results demonstrated that as pomegranates mature, their polyphenol concentration, content, and color intensity increase. Phenolic content was found to vary by geographic location, with El Jahliye showing low to moderate levels, Rachiine showing moderate to high levels, and Hasbaya exhibiting all three polyphenol levels. These variations in maturity and pomegranate composition may be influenced by many factors besides climate including soil, genetics, region, storage conditions, and harvesting method. We proposed optimal harvesting dates based on phenolic and technological maturity, though these dates did not fully align with the actual harvesting times, suggesting more awareness campaigns for the farmers to be implemented. Regarding AI models, the MLP model successfully predicted pomegranate acidity and categorized pomegranates based on their polyphenol content. Additionally, the logistic regression model outperformed the MLP model in predicting the technological maturity of pomegranates and their polyphenol categorization, demonstrating greater reliability and accuracy. Finally, this work allowed us to determine optimal harvesting dates based on technological and phenolic maturity indicators. Further, our AI models demonstrate promising results in estimating both technological maturity and polyphenol content in Lebanese pomegranates based on simple physical, geographical, and environmental features. Our future research will focus on examining the effects of weather and soil on pomegranate ripening and raising awareness of technological and phenolic maturity to guide optimal harvesting. We aim to improve the performance of the AI model by increasing the sample size and measuring anthocyanin content to link it to color intensity. Besides, we aim to employ convolutional neural networks to train the model on the collected photos of pomegranate correlating visual features with polyphenol levels. This work will also be extended to a smartphone app for non-destructive estimation of polyphenol content, helping farmers optimize pomegranate quality.

## Data Availability

The datasets used and/or analyzed during the current study are available from S.A. on reasonable request.
